# Phase II, Open Label, Randomized Comparative Trial of Ondansetron Alone versus the Combination of Ondansetron and Aprepitant for the Prevention of Nausea and Vomiting in Patients with Hematologic Malignancies Receiving Regimens Containing High-Dose Cytarabine

**DOI:** 10.1155/2015/497597

**Published:** 2015-01-15

**Authors:** Talha Badar, Jorge Cortes, Gautam Borthakur, Susan O'Brien, William Wierda, Guillermo Garcia-Manero, Alessandra Ferrajoli, Tapan Kadia, Rebeca Poku, Hagop Kantarjian, Gloria Mattiuzzi

**Affiliations:** Department of Leukemia, MD Anderson Cancer Center, 1515 Holcombe Boulevard, Unit 428, Houston, TX 77030, USA

## Abstract

*Background*. Aprepitant is a P/neurokinin-1 receptor antagonist approved for the prevention of CINV in moderate emetic risk chemotherapy. We explored its effectiveness in patients with leukemia receiving cytarabine-based chemotherapy.* Methods*. Patients were randomized to ondansetron (OND) 8 mg IV 30 minutes before cytarabine followed by 24 mg IV continuous infusion daily until 6–12 hours after the last dose of chemotherapy alone or with aprepitant (APREP) oral 125 mg 6–12 hrs before chemotherapy and 80 mg daily until 1 day after the last dose of chemotherapy.* Results*. Forty-nine patients were enrolled in each arm; 42 in OND and 41 in OND + APREP arm were evaluable for efficacy. The ORR with OND + APREP was 80% compared to 67% with OND alone (*P* = 0.11). On days 6 and 7, higher proportion of patients treated with OND + APREP were free from nausea (74%, 74% versus 68%, 67%; *P* = 0.27 and 0.18, resp.). Requirement of rescue medications on days 2 and 3 was fewer in OND + APREP arm 7% and 5% compared to 21% and 16% in the OND arm, respectively (*P* = 0.06 and *P* = 0.07).* Conclusions*. There was a trend for overall improvement in emesis with ondansetron plus aprepitant. The potential benefit of this approach with specific chemotherapy combinations remains to be determined.

## 1. Introduction

Chemotherapy-induced nausea and vomiting (CINV) can be a significant problem for patients, associated with deterioration in the quality of life and decline of physical and cognitive functions, and may ultimately affect the patient's desire to continue further chemotherapy [1, 2]. Among the proposed mechanisms responsible for chemotherapy-induced vomiting is the local or systemic release of neurotransmitters secondary to cellular injury induced by chemotherapy. The major excitatory neurotransmitters that are involved in emesis are 5-hydroxytryptamine (serotonin) and dopamine [3]. Most of the available antiemetic drugs act on a single pathway. Although this might be sufficient in many instances, a combination of antiemetic drugs is required to get the best antiemetic response in patients receiving moderate emetogenic chemotherapy [4].

Cytarabine-containing regimens have been the backbone for the treatment of acute myeloid leukemia (AML) for the last four decades. Cytarabine is classified as one of the “moderate emetic risk chemotherapy agents,” in the American Society of Clinical Oncology (ASCO) and NCCN classification of the emetic risk [5]. When cytarabine is used in combination with other chemotherapeutic agents, nausea and vomiting can worsen substantially.

Although the combination of a 5-HT3 antagonist plus corticosteroids is the recommended antiemetic prophylaxis for patients receiving moderate emetic risk chemotherapy regimen, a significant subset of patients still experience acute and/or delayed chemotherapy-induced nausea and vomiting (CINV) [6]. More than 50% of the patients with AML still require rescue medication for nausea, with standard antiemetic prophylaxis [7]. Thus, 5-HT3 inhibitors alone are not sufficient to prevent nausea in patients with AML receiving multiple day combination chemotherapy, particularly on days when the acute and delayed emetic effect of the combination chemotherapy agents overlap (typically days 3 to 5).

Aprepitant is a substance P/neurokinin-1 (NK1) receptor antagonist approved for use in combination with a 5-HT3 receptor antagonist and dexamethasone for the prevention of acute and delayed CINV [8, 9]. Studies in patients with solid tumors receiving moderately or highly emetogenic multiday chemotherapy have demonstrated that aprepitant is effective in preventing CINV during the first 24 hrs after chemotherapy and also provides protection against delayed CINV [10–16]. Here we present the first study of aprepitant for the management of nausea associated with cytarabine-based chemotherapy in patients with leukemia. We hypothesized that addition of aprepitant to ondansetron will improve emetic symptoms in patients receiving high-dose cytarabine-based multiday chemotherapy for leukemia.

## 2. Material and Methods

### 2.1. Patients

Adult patients (≥18 years old) with AML, high-risk MDS, or chronic myeloid leukemia (CML) in blast phase receiving cytarabine-based chemotherapy at a dose of ≥1 g/m^2^/day for at least 3 days were eligible for this study. Exclusion criteria included patients with emesis or grade 2 or 3 nausea presenting within 24 hours before the start of chemotherapy, ongoing emesis due to any organic etiology, and known hypersensitivity to the study drug or to 5-HT3 receptor antagonists and patients receiving pimozide, terfenadine, astemizole, or cisapride due to possible drug-drug interactions. The protocol was approved by the institutional review board and all patients gave written informed consent.

Patients were randomized to Arm (1) ondansetron (OND) 8 mg IV 30 minutes before cytarabine followed by ondansetron 24 mg IV continuous infusion daily until 6–12 hours after the end of last chemotherapy infusion or Arm (2) aprepitant (APREP) 125 mg PO plus ondansetron 8 mg IV 30 minutes before cytarabine followed by ondansetron 24 mg IV continuous infusion daily until 6–12 hours and aprepitant 80 mg oral daily until 1 day after the end of last chemotherapy. Patients were followed for 72 hrs after chemotherapy. A diary was used to record daily number of episodes of nausea and/or vomiting experienced during the study and to record any need for rescue medications for nausea. Patients were followed for a total of 6 days, starting from first day of chemotherapy.

Complete response (CR) was defined as no episodes of emesis and no use of rescue antiemetics during the study period. Partial response (PR) was defined as ≤1 episode of emesis, no use of rescue medication, and no more than grade 2 nausea as defined by the National Cancer Institute Common Terminology Criteria for Adverse Events (version 3.0) during the entire study period. Time to treatment failure was defined as the time to first emetic episode or first need of rescue medication, whichever occurred first. Patients were removed from the study if they developed grade 4 nonhematological toxicity related to ondansetron or aprepitant and due to noncompliance with study medications or attendance to schedule visits.

### 2.2. Statistical Considerations

This was an open-label, randomized phase II study of ondansetron alone (control) versus combination of ondansetron and aprepitant (experimental) for the prevention of nausea and vomiting in patients with hematologic malignancies receiving high-dose cytarabine-containing chemotherapy. The primary endpoint of the study was the prevention of emesis and avoidance of rescue medication during the administration of chemotherapy.

Historical data suggest that about 40% of the patients receiving ondansetron only would achieve success. A maximum of 100 patients were planned to be enrolled in the study aiming to detect a 15% improvement in success rate with the experimental arm. Patients were randomized 1 : 1 to the two treatment arms. Bayesian monitoring for futility was implemented after the 15th patient was enrolled and had been evaluated for complete response. Categorical data were tabulated by frequency and percentage; continuous variables were summarized using descriptive statistics (mean, standard deviation, and median range). Chi square and Fisher exact test were used to assess the level of significance between variables and response. *P* value below 0.05 was considered statistically significant.

## 3. Results

### 3.1. Patient Baseline and Demographic Characteristics

Ninety-eight patients were registered in the study, 49 to each arm. Among them 83 were evaluable for efficacy, 42 in the OND arm and 41 in the OND + APREP arm. Fifteen patients were excluded from the analysis: 4 patients had nausea/vomiting prior to chemotherapy, 5 received ondansetron ≥30 minutes prior to cytarabine, 2 received prohibited concomitant medications with possible drug-drug interactions, 1 patient had grade 3 GI toxicity, thought to be related to study drug, 1 patient had prolonged QTc interval, 1 patient withdrew consent, and 1 patient had a dose reduction of cytarabine below 1 gm/m^2^. Patients who received at least one dose of the study drugs and had adverse events were included in the safety analysis. The treatment groups were well balanced in their pretreatment characteristics (Table 1).

### 3.2. Efficacy

The response to study drug is presented in Table 2. There was a trend for a higher overall response rate (complete plus partial responses) for patients in the OND + APREP arm but the difference was not statistically significant (OND 67%, OND + APREP 80%; *P* = 0.11). This correlated with a lower rate of treatment failure in the OND + APREP arm than in the OND arm (OND 33%, OND + APREP 19%; *P* = 0.11). We then evaluated the response to antiemetic therapy by day of therapy throughout the 6 days of study evaluation. The proportion of patients that remained free from nausea remained mostly constant. Approximately 70% to 75% of patients in both arms were free of nausea on days 1 through 4. On days 5 and 6 there was a trend for a higher proportion of patients in the OND + APREP arm (76% and 74%) that were free from nausea than in the OND arm (68% and 67%, resp.; *P* = 0.27 for day 5 and 0.34 for day 6) (Table 3).

In the intention-to-treat analysis all patients who received even a single day of treatment on protocol were included. The ORR in the OND + APREP arm was 70% compared to 58% in OND arm; *P* = 0.369. The CR rate was similar in both arms: 44% in the OND arm and 46% in the OND + APREP arm; *P* = 1.0. Partial responses were more frequent in the OND + APREP arm (23%) than in the OND arm (14%). This corresponded to a treatment failure rate of 42% in the OND arm and 30% in the OND + APREP arm; *P* = 0.369.

### 3.3. Rescue Medication

**Figure 1:**
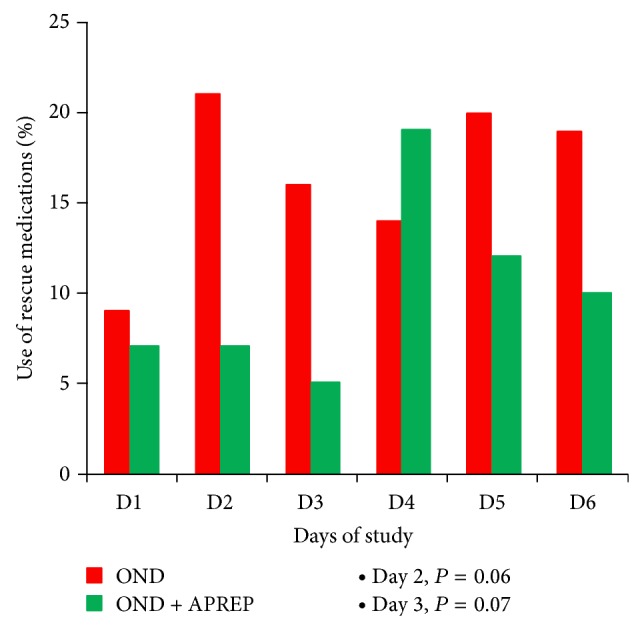
Proportion of patients who required rescue medications.

Overall 36% of patients required rescue medications at some time during the course of the study period. This included 38% in the OND arm and 34% in the OND + APREP arm. Overall, 14% of patients required rescue medications more than once, 19% in the OND arm and 10% in the OND + APREP arm. Rescue medication was seldom required on day 1 in both arms. Requirements for rescue medications subsequently increased more rapidly in the OND arm, such that on days 2 and 3 significantly fewer patients in the OND + APREP arm (7% and 5%) required rescue medication compared to the OND arm (21% and 16%; *P* = 0.06 and *P* = 0.07, resp.) (Table 4, Figure 1).

### 3.4. Adverse Events

In both arms 87 patients were evaluated for adverse events who had even received a single dose of study drug: 43 (49%) in the OND (Arm 1) and 44 (51%) in the OND + APREP (Arm 2). Adverse events were mainly grades 1 and 2 with comparable incidences in both arms. The most common adverse events of all grades overall observed were diarrhea in 23 (26%: Arm 1, 11 [26%] and Arm 2, 12 [27%]), headache in 22 (25%: Arm 1, 11 [26%] and Arm 2, 11 [25%]), fatigue in 18 (21%: Arm 1, 9 [21%] and Arm 2, 9 [20%]), and constipation in 17 (19%: Arm 1, 7 [16%] and Arm 2, 10 [23%]) patients. The grades 3-4 toxicities seen overall were headache in 2 (5%) patients, only in Arm 2, and diarrhea in 3 (3%: Arm 1, 1 [2%] and Arm 2, 2 [5%]) patients and grade 3 syncope seen in 1 patient, Arm 1. Other less frequent side effects were indigestion, abdominal pain, dizziness, and edema (Table 5).

## 4. Discussion

To the best of our knowledge, this is the first prospective randomized trial of aprepitant with standard antiemetic for prevention of CINV in patients with AML or high-risk MDS receiving intermediate or high-dose cytarabine-based chemotherapy. The experimental arm (aprepitant plus ondansetron) had a trend for a higher overall response rate. The difference however was not statistically significant, perhaps because of the small sample size. Alternatively, the inclusion of patients treated mostly with a moderately emetogenic chemotherapy regimen (rather than a highly emetogenic regimen) may have also affected the outcome. The difference was most notable on days 6 and 7, with a decrease in the requirements for rescue medication on days 2 and 3.

Chemotherapy-induced nausea and vomiting in the patients with hematological malignancies had received little attention to date with few studies addressing this issue. Intermediate/high-dose cytarabine (≥1 gm/m^2^) is considered to have moderate emetic risk according to recent oncology guidelines, with a 30% to 90% risk of emesis without the use of prophylactic antiemetics [5]. In spite of using prophylactic antiemetics as per guidelines, patient receiving multiday, high-dose cytarabine chemotherapy for hematological malignancies, the emesis control is not optimum [7, 15]. In trying to address this problem, we recently reported [7] a prospective study comparing 2 schedules of palonosetron versus ondansetron, two different 5-HT3 inhibitors, for CINV in patient with AML receiving high-dose cytarabine. Patients were randomized to ondansetron 8 mg loading dose before chemotherapy followed by 24 mg continuous infusion daily until 12 hours after the last dose of cytarabine, palonosetron 0.25 mg 30 minutes before chemotherapy daily from day 1 of high-dose cytarabine up to day 5, or palonosetron 0.25 mg 30 minutes before high-dose cytarabine on days 1, 3, and 5. The overall response rate (ORR) in the ondansetron arm was 34%, in the palonosetron on days 1, 3, and 5 44% and in the palonosetron on days 1–5 46%. Complete response rates were 21%, 35%, and 31%, respectively (*P* = 0.46). On days 6 and 7 more patients receiving palonosetron on days 1–5 were free from nausea (*P* = 0.001 and 0.024, resp.). These results suggest that palonosetron might be more effective in this setting although the small sample size made the differences not statistically significant.

In another attempt at improving the antiemetic regimens for AML, Uchida and colleagues [15] published a retrospective analysis of patients with hematological malignancies, receiving multiday chemotherapy. The response rate in preventing emesis was compared between granisetron alone versus granisetron and aprepitant combination. All patients received 3 mg of granisetron 30 minutes before chemotherapy administration. Patient in the aprepitant group received 125 mg of aprepitant orally before chemotherapy administration followed by 80 mg orally on day 2 to day 5, in addition to granisetron. Complete response was 76% in the aprepitant arm versus 50% in the control group (*P* value = 0.013).

In our study the overall response rate was 80% in the APREP + OND group and 67% in OND alone group (*P* value = 0.11). The proportion of patients with complete responses was 48% in OND group versus 51% in OND + APREP group (*P* value = 0.45). The overall response was higher than that reported by Mattiuzzi et al. [7] but lower than that in the study by Uchida et al. [15]. This discrepancy may be related to differences in the underlying disease, the patient population, and the chemotherapy regimens the patients received; thus comparisons should be made with caution. Still the response rate appears higher than that in the study conducted by Mattiuzzi et al. [7] probably because we used a combination of ondansetron with aprepitant rather than a single agent. In the study by Uchida et al. [15] only 17 out of 42 had AML and among them 13 patients receive high-dose cytarabine (≥1 gm/m^2^). Among those who received high-dose cytarabine, the complete response rate was 30% which is lower than the responses achieved with antiemetics in patients receiving chemotherapy other than high-dose cytarabine. One more significant difference in Uchida et al. study compared to ours is that patients received granisetron instead of ondansetron. In addition, in our present study all patients received high- or intermediate-dose cytarabine in combination with idarubicin, fludarabine, or clofarabine which may add to the emetogenic potential of the chemotherapy.

In our study, with ondansetron, more than 75% of patients were free from nausea on day 1 and day 2. This fraction decreased from day 3 to day 7. This suggests that the chemotherapy effect might be cumulative and the antiemetic benefit lost to some extent with additional doses of chemotherapy. In contrast, with aprepitant plus ondansetron patients appeared to remain free from nausea and required less rescue medications throughout the observation period. A similar trend was seen in the study by Mattiuzzi et al. [7], where patient who received 5 days of palonosetron had less nausea on day 6 and day 7. The results of our study support the hypothesis that aprepitant is effective in decreasing the incidence of delayed nausea with a more sustained antiemetic effect.

The combination of aprepitant and ondansetron was well tolerated. Adverse effects were mainly grades 1 and 2: headache, diarrhea, fatigue, constipation, and indigestion. Observed adverse effect profile is similar to the reported literature [11, 13]. One patient in the OND arm had grade 3 diarrhea and two patients each had grade 3 diarrhea and headache in the OND + APREP arm. One patient in the OND arm had grade 3 syncope.

In conclusion, the combination of ondansetron and aprepitant provides adequate antiemetic therapy for patient receiving multiday moderate emetic risk chemotherapy. However, a more definitive study with larger numbers of patients is needed to confirm these observations. Alternative combinations (e.g., combinations including dopamine antagonists) should also be investigated.

## Figures and Tables

**Table 1:** Baseline characteristics.

	Ondansetron; Arm 1 *N* = 49 *N* (%)	Ondansetron + aprepitant; Arm 2 *N* = 49 *N* (%)	*P*
Sex			
Women	21 (43)	22 (45)	0.53
Men	28 (57)	27 (55)	
Race/ethnicity			
Caucasian	40 (83)	37 (76)	0.24
Hispanic	3 (6)	6 (12)	0.62
African American	5 (10)	4 (8)	0.50
Asian	0 (0)	2 (4)	0.50
Median age, y (range)	53 (30–68)	49 (21–70)	
Diagnosis			
AML	47 (96)	47 (96)	0.69
MDS	1 (2)	2 (4)	0.50
CMML	1 (2)	0 (0)	0.50
Ind. chemotherapy	44 (90)	38 (78)	0.08
Sal. chemotherapy	5 (10)	11 (22)	
Chemotherapy agents used in combination with cytarabine			
Idarubicin	7 (14)	7 (14)	0.61
Fludarabine	10 (20)	8 (16)	0.30
Idarubicin + clofarabine	24 (49)	24 (49)	0.50
Clofarabine, idarubicin, and decitabine	1 (2)	1 (2)	0.75
Investigational drug + idarubicin	4 (8)	7 (14)	0.50
GO + fludarabine	2 (4)	1 (2)	0.50
Infection at baseline	10 (20)	9 (18)	0.45
Use of antibiotic at baseline	9 (18)	9 (18)	0.56

Ind: induction, Sal: salvage, and GO: gemtuzumab ozogamicin.

**Table 2:** Responses to antiemetic therapy.

Response	Ondansetron; Arm 1 Number (%)	Ondansetron + aprepitant; Arm 2 Number (%)	*P*
Overall response	28 (67)	33 (80)	0.11
Complete response	20 (48)	21 (51)	0.45
Partial response	8 (19)	12 (29)	0.20
Failure	14 (33)	8 (19)	0.11

**Table 3:** Proportion of patients free from nausea from day 1 to day 6.

Nausea-free	Ondansetron; Arm 1 Number (%)	Ondansetron + aprepitant; Arm 2 Number (%)	*P*
Day 1	35 (78)	32 (74)	0.45
Day 2	35 (78)	32 (74)	0.45
Day 3	34 (77)	30 (71)	0.35
Day 4	32 (73)	30 (71)	0.54
Day 5	30 (68)	32 (74)	0.27
Day 6	29 (67)	31 (74)	0.18

**Table 4:** Proportion of patients requiring rescue medications from day 1 to day 6.

Use of rescue medications	Ondansetron; Arm 1 Number (%)	Ondansetron + aprepitant; Arm 2 Number (%)	*P*
Day 1	4 (9)	3 (7)	0.52
Day 2	9 (21)	3 (7)	0.06
Day 3	7 (16)	2 (5)	0.07
Day 4	6 (14)	8 (19)	0.34
Day 5	8 (20)	5 (12)	0.30
Day 6	8 (19)	4 (10)	0.18

**Table 5:** Adverse events.

Adverse events	*N* (%) with any grade			*N* (%) with grades 1-2		*N* (%) with grades 3-4		
	Overall	OND	OND + APR	OND	OND + APR	Overall	OND	OND + APR
Diarrhea	23 (26)	11 (26)	12 (27)	10 (23)	11 (25)	3 (3)	1 (2)	2 (5)
Headache	22 (25)	11 (26)	11 (25)	11 (26)	9 (20)	2 (2)	0 (0)	2 (5)
Fatigue	18 (21)	9 (21)	9 (20)	9 (21)	9 (20)	0 (0)	0 (0)	0 (0)
Constipation	17 (19)	7 (16)	10 (23)	7 (16)	10 (23)	0 (0)	0 (0)	0 (0)
Indigestion	9 (10)	3 (7)	6 (14)	3 (7)	6 (14)	0 (0)	0 (0)	0 (0)
Edema	8 (9)	3 (7)	5 (11)	3 (7)	5 (11)	0 (0)	0 (0)	0 (0)
Abdominal pain	5 (6)	2 (5)	3 (7)	2 (5)	3 (7)	0 (0)	0 (0)	0 (0)
Dizziness	3 (3)	1 (2)	2 (5)	1 (2)	2 (5)	0 (0)	0 (0)	0 (0)
Syncope	1 (1)	1 (2)	0 (0)	0 (0)	0 (0)	1 (1)	1 (2)	0 (0)
Hypotension	1 (1)	0 (0)	1 (2)	0 (0)	1 (2)	0 (0)	0 (0)	0 (0)
